# Perfluoroalkylated Substances (PFAS) Associated with Microplastics in a Lake Environment

**DOI:** 10.3390/toxics9050106

**Published:** 2021-05-11

**Authors:** John W. Scott, Kathryn G. Gunderson, Lee A. Green, Richard R. Rediske, Alan D. Steinman

**Affiliations:** 1 Illinois Sustainable Technology Center, Prairie Research Institute, University of Illinois, Champaign, IL 61820, USA; kggunde2@illinois.edu (K.G.G.); leegreen@illinois.edu (L.A.G.); 2 Annis Water Resources Institute, Grand Valley State University, Muskegon, MI 49441, USA; redisker@gvsu.edu (R.R.R.); steinmaa@gvsu.edu (A.D.S.)

**Keywords:** per- and polyfluoroalkyl substances, microplastics, Muskegon Lake

## Abstract

The presence of both microplastics and per- and polyfluoroalkyl substances (PFAS) is ubiquitous in the environment. The ecological impacts associated with their presence are still poorly understood, however, these contaminants are extremely persistent. Although plastic in the environment can concentrate pollutants, factors such as the type of plastic and duration of environmental exposure as it relates to the degree of adsorption have received far less attention. To address these knowledge gaps, experiments were carried out that examined the interactions of PFAS and microplastics in the field and in a controlled environment. For field experiments, we measured the abundance of PFAS on different polymer types of microplastics that were deployed in a lake for 1 month and 3 months. Based on these results, a controlled experiment was conducted to assess the adsorption properties of microplastics in the absence of associated inorganic and organic matter. The adsorption of PFAS was much greater on the field-incubated plastic than what was observed in the laboratory with plastic and water alone, 24 to 259 times versus one-seventh to one-fourth times background levels. These results suggest that adsorption of PFAS by microplastics is greatly enhanced by the presence of inorganic and/or organic matter associated with these materials in the environment, and could present an environmental hazard for aquatic biota.

## 1. Introduction

Per- and polyfluoroalkyl substances (PFAS) have received considerable attention from the scientific community and regulatory agencies. By nature of design, these compounds are thermally stable, oxidatively recalcitrant, and resist microbial degradation [1,2,3]. Bioaccumulation of legacy PFAS that was released into the environment has been observed in organisms at various trophic levels, such as phytoplankton, fish, porpoise, and polar bears [4,5,6,7]. Large knowledge gaps exist regarding bioavailability, bioaccumulation, and biotransformation of legacy and residual PFAS, particularly in lower-trophic level freshwater organisms, which may influence PFAS exposure to humans via fish-based consumption.

Plastic in the environment is also persistent, and rather than biodegrade, macroplastics (≥5 mm) erode into microplastics (<5 mm) via physical and chemical processes and exposure to ultraviolet light [8]. Primary microplastics can also enter the environment through the loss of pre-production plastic pellets during manufacturing or transport, and more recently, wastewater effluent has been identified as a source of microbeads originating from cosmetic products and microfibers shed from clothing and textile laundering [9,10].

Certain persistent organic pollutants (POPs) are known for their carcinogenic, endocrine-disrupting, and reproductive effects [11]. In addition, POPs adsorb to plastics at concentrations greater than the surrounding environment and become biologically available for absorption after ingestion [12]. The bioaccumulation of plastic-borne POPs is prevalent in sea bird populations, for example, where the mass of plastic ingested by short-tailed shearwaters is correlated with polychlorinated biphenyl (PCB) body burden [13]. In the Great Lakes region, the bioaccumulation of polyaromatic hydrocarbons in salmonids was cited as a likely cause of thyroid deficiencies and goiter in wild herring gulls (*Larus argentatus*) and in lab rats sustained on a diet consisting of Great Lakes coho salmon (*Oncorhynchus kisutch*), suggesting that predation is a pathway for the bioaccumulation of POPs in the Great Lakes food web [14]. It is critical to identify routes of human exposure to PFAS because they have been detected in human blood and breast milk [15,16,17]. In addition to drinking water, diet may be a major exposure pathway for humans [17,18]. In the U.S., national fish monitoring studies suggested that fish consumption may be a source of human exposure to PFAS because these compounds have been frequently detected in fish tissues collected from the Great Lakes and urban rivers across the country [19,20]. In addition, PFAS have been found in shrimp and seafood [21,22]. In the aquatic environment, bioaccumulation from different media and organisms (i.e., water, sediment, phytoplankton, and fish) is well known as a major mechanism for PFAS transfer to the food chain [23]. PFAS is of special concern in Michigan, where some of the highest groundwater concentrations have been detected [24], and there are concerns about these plumes contaminating surface waters.

Like many of the chemicals known to sorb to plastics, PFAS have properties that can facilitate the potential of microplastics to serve as their carriers [25]. To the best of our knowledge, no previous studies have been conducted to investigate the nature and concentrations of PFAS adsorbed to microplastics in the environment. Another factor influencing the adsorption of chemicals to plastics is the role of biofilms, a consortium of algae, bacteria, and other microorganisms that can affect the fate and level of impact of adsorbed contaminants within freshwater systems [26]. Given the prevalence of PFAS and microplastics in natural waters, coupled with the extremely long persistence time of both classes of pollutants, these two groups of emerging contaminants may act synergistically in food webs to cause adverse effects in fish and wildlife, as well as humans.

Our study was designed to address this knowledge gap with experiments that examined the interactions of PFAS and microplastics in the field and in a controlled environment. For field experiments, we examined the abundance of seven common PFAS on three different polymer types of microplastics that were deployed in a lake over a time period of 1 and 3 months. Aqueous samples were also collected and analyzed at the time of deployment to serve as the background concentration of PFAS. Finally, based on the results of the field-based microplastic experiment, we conducted a controlled, lab-based experiment with the most abundant PFAS measured from the field experiment to assess the adsorption properties of microplastics in the absence of associated organic/inorganic matter and biofilm.

## 2. Materials and Methods

**Figure 1:**
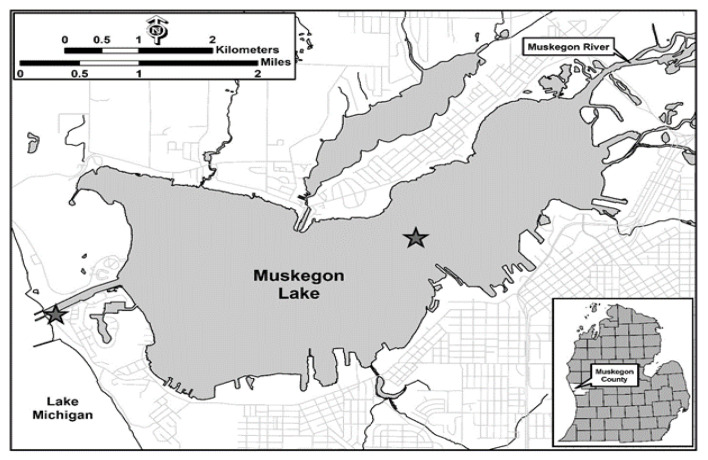
Locations (channel and lake) for Deployment (filled stars) of Microplastics in Muskegon Lake.

Microplastic Deployment (Field Study): Plastic materials were deployed at two sites located in Muskegon Lake, Michigan (Figure 1). The deployed materials included low-density polyethylene (LDPE), polypropylene (PP), and polyethylene terephthalate (PET), which were 2 to 4 mm in size, and incubated in separate containers (see below).

For lake deployment of the microplastics, incubation tubes were constructed and mounted to a deployment frame. Each tube contained approximately 42 g of each plastic type and each frame contained 3 polymer types with 4 replicates per frame. Therefore, a total of 12 tubes were randomly arranged on each frame. All frames were deployed on 4 June 2018. One of the sites was centrally located in mesotrophic Muskegon Lake (43.23834 N, 86.27923 W; depth = 12 m) and was placed at the water-sediment interface (Lake Bottom); this site was adjacent to the Muskegon Lake Observatory, which collects water quality data throughout the water column on a near-continuous basis (https://www.gvsu.edu/wri/buoy/, accessed on 10 May 2021). The other site chosen was near the sea wall at the more oligotrophic Lake Michigan–Muskegon Lake navigation channel (43.22769 N, 86.33911 W; depth = 2 m and 4 m). For the channel site, a frame was placed at a depth of 2 m and another at the sediment–water interface (channel water column and channel bottom, respectively). Incubation times were for 1 and 3 months and a total of 36 tubes were used. Aqueous samples were collected at the time of initial deployment and considered the background concentration of PFAS at these sites. In addition, water quality data including water temperature, conductivity, and dissolved oxygen were recorded during retrieval of the deployment racks at their respective timepoints (see Supplemental Table S1). Further details regarding sample deployment and treatment are published elsewhere [27].

Controlled PFAS Exposure (Laboratory Study): The three most abundant PFASs from the field study (PFOA—perfluorooctanoic acid, PFHxA—perfluorohexanoic acid, and PFHpA—perfluoroheptanoic acid) were added to flasks containing 50 mL of deionized water. The exposure solution was prepared at a concentration of 5 μg/L for each PFAS. Ten grams each of fresh, non-incubated plastic type were added to the flasks. The solutions with microplastics were then placed in a laboratory incubator and shaken at 90 revolutions per minute (RPM) at room temperature for 1 month. After that time, the microplastics were collected by filtration (Whatman, Glass Microfibre (GF/F), pore size: 0.7 µm).

Sample Preparations and Analysis of PFAS: Sample preparation and analysis of PFAS was performed by US EPA Method 537 [28]. Isotopically enriched PFAS were spiked into all test materials to serve as surrogates for the native PFAS.

Pristine (non-incubated—laboratory study) and incubated (field study) microplastics were prepared by a solid-liquid extraction method utilizing a 10 g sample and methanol as an extraction solvent (3 × 20 mL). The pooled organic fractions were then concentrated to 1.0 mL before analysis.

Seven individual PFAS were targeted for field samples since they are the most abundant PFAS previously detected in the Great Lakes [29]. These PFAS compounds were perfluorobutanesulfonic acid (PFBSPFHxA, PFHpA, PFOA, perfluorononanoic acid (PFNA), perfluorodecanoic acid (PFDA), and perfluorooctanesulfonic acid (PFOS). This field study served as a “screening tool” for which PFASs were most relevant for a controlled experiment and based on these results, the laboratory study focused on PFOA, PFHxA, and PFHpA. PFAS compounds were analyzed by liquid chromatography tandem mass spectrometry (LC-MSMS) using a Waters Alliance 2695 coupled to a Quattro Micro tandem mass spectrometer (Waters Corporation, Milford, MA, USA).

Quality control parameters associated with the samples included reagent blanks, reagent blank spikes, and matrix spikes. Reagent blanks contained all the materials used for sample preparations and reagent blank spikes were similar yet contained the target PFAS. Matrix spikes were prepared by spiking a duplicate sample with PFAS.

All final PFAS results were calculated by the isotope dilution method, which utilizes the isotope surrogate and corrects the native PFAS concentrations based on their recoveries. Reported results reflect the average of multiple sample preparation and analysis. The associated errors for these results were derived from either the relative percent difference (%RPD) or relative standard deviation (%RSD) of the multiple measurements. In situations where a target PFAS was detected in one replicate but not others, the value for the single result is reported.

Data Analysis—Field Study: Summed PFAS concentrations (when reported above minimum detection levels) were statistically analyzed separately for each deployed microplastic substrate using 2-way analysis of variance (ANOVA) to determine whether deployment site (channel water column, channel bottom, lake bottom), deployment duration (1 month, 3 months), or the interaction between site and duration had a significant effect on post-incubation PFAS concentrations. Each combination of site and duration factors had *n* = 2 tube replicates for each of the 1 month and 3 month sampling events. ANOVA assumptions of normality and equal variance were tested with Shapiro–Wilk and Brown–Forsythe tests, respectively. However, 2-way ANOVAs for each microplastic substrate violated assumptions of equal variance (i.e., Brown–Forsythe: *p* > 0.05), which were not improved by data transformation, and are presented herein using untransformed data. When 2-way ANOVAs detected significant differences, post hoc multiple comparisons were made using Holm–Sidak tests. A 1-way ANOVA was used to determine whether the plastic type (polypropylene, polyethylene, polyester) influenced final microplastic PFAS concentrations (*n* = 3 replicates per plastic type).

Data Analysis—Laboratory Study: Summed PFAS concentrations (PFHxA, PFHpA, and PFOA) were analyzed using 1-way ANOVA to determine whether plastic type (polypropylene, polyethylene, polyester) influenced final microplastic PFAS concentrations. Each microplastic type had *n* = 3 independent sample replicates. Assumptions of normality and variance were tested as described above and detected no violations and data were not transformed. Post hoc multiple comparison was completed using a Tukey test. All statistical analyses were completed using Sigma Plot (v14.0).

## 3. Results

Field Study: None of the seven target PFAS were detected above the detection limit for the trip blank, reagent blanks, and pristine (non-incubated) microplastics. This indicates that the sample collection, sample preparation techniques, and starting materials were free from PFAS contamination.

Unless otherwise stated, all PFAS concentrations are reported as a sum of the seven PFAS measured in the field study or the three PFAS in the laboratory study. The concentrations of PFASs measured from the field water samples were 2.8 ng/L (RPD = 16%) and 3.3 ng/L (RPD = 4.2%) in the channel and lake, respectively. PFOA, PFHpA, PFBS, and PFOS were detected in these samples, with PFOA at the greatest concentration. These results were considered the background concentration of PFAS to which the deployed microplastics were exposed.

**Figure 2:**
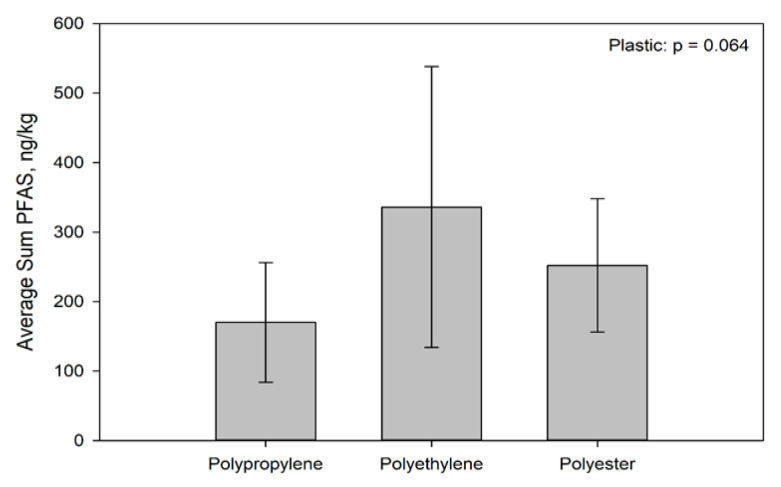
Average Sum of 7 PFAS (ng/kg) by Plastic Types for Materials Deployed in Muskegon Lake, MI for 1 Month and 3 Month Incubations in the Environment.

PFAS concentrations associated with the plastics (including inorganic and organic matter associated with them) after incubation in Muskegon Lake ranged from 67 ng/kg to 730 ng/kg. These materials concentrated PFASs by factors ranging from 24 to 259 times the background aqueous concentration in the lake water within 1 to 3 months. Figure 2 presents the average PFAS by plastic type only, irrespective of location or exposure duration. The trend from lowest to highest concentrator is polypropylene < polyester < polyethylene. However, these differences were only marginally significant (*p* < 0.10) due to the high variance among plastics.

**Figure 3:**
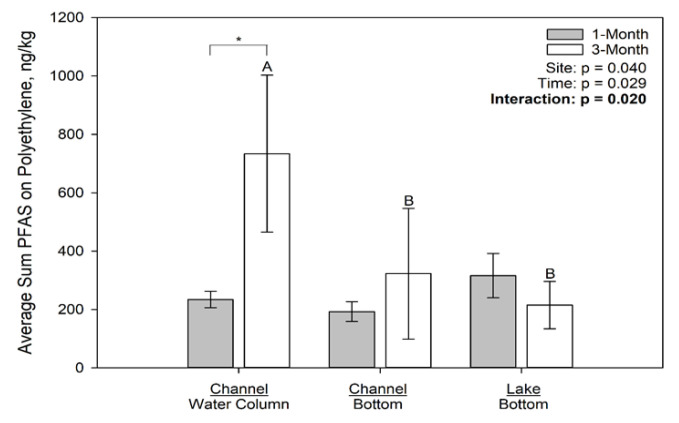
Average Sum of 7 PFAS (ng/kg) on Polyethylene Deployed at Different Locations in Muskegon Lake, MI. Different letters among bars indicate statistically significant differences among sites for either the 1 month or 3 month incubation period. Asterisks indicate statistically significant differences between the 1 month vs. 3 month incubation at a specific site.

**Figure 4:**
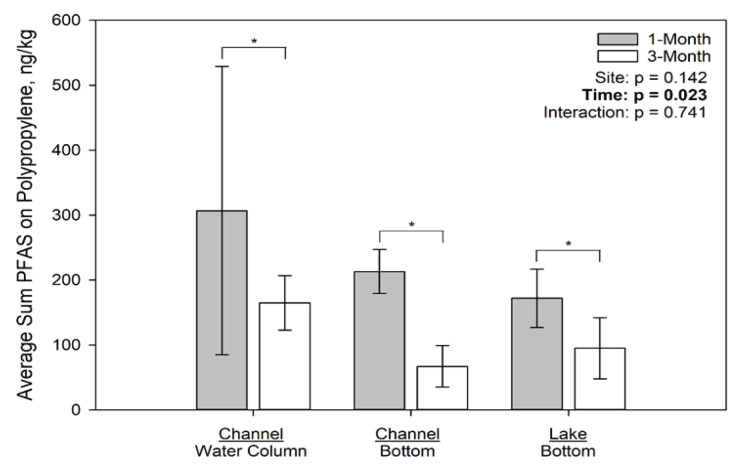
Average Sum of 7 PFAS (ng/kg) for Polypropylene Deployed in Muskegon Lake, MI. Asterisks indicate statistically significant differences between the 1 month vs. 3 month incubation at a specific site.

**Figure 5:**
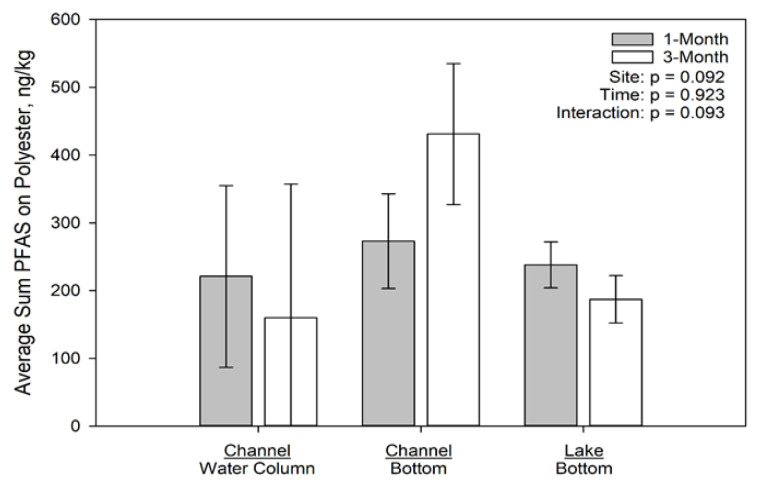
Average Sum of 7 PFAS (ng/kg) for Polyester Deployed in Muskegon Lake, MI.

The concentrations of PFAS associated with the deployed microplastics by location and time are presented in Figure 3, Figure 4 and Figure 5 for polyethylene, polypropylene, and polyester, respectively. On polyethylene (Figure 3), PFAS concentrations were not significantly different among sites at 1 month but were significantly different at 3 months due to concentrations on the plastics at the channel water column site exceeding those at both the channel bottom and lake bottom sites. On polypropylene (Figure 4), only time had a significant effect on PFAS concentration, with the 1 month concentrations greater than the 3 month concentrations; neither site nor the interaction term were statistically significant. Finally, on polyester (Figure 5), PFAS concentrations were not significantly affected by time or site.

**Figure 6:**
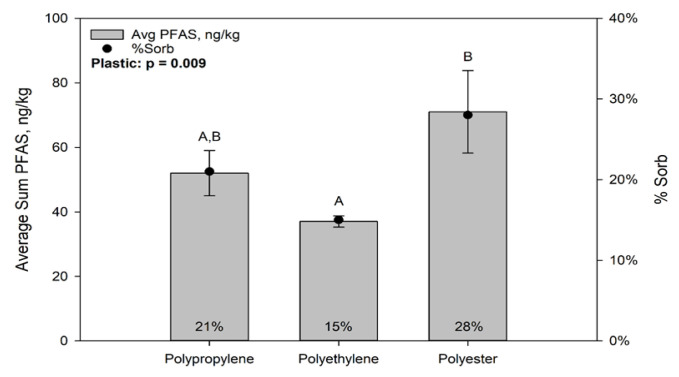
Average Summed 3 PFAS concentration (ng/kg) and Percent Adsorption (number above each bar) of PFAS on Plastic for Laboratory Study. Different letters among bars indicate statistically significant differences among sites for either the 1 month or 3 month incubation period.

Laboratory Study: PFHxA, PFHpA, and PFOA were the most abundant PFAS associated with the microplastics incubated at the lake sites, so these 3 were the focus of the laboratory experiments. Figure 6 presents the average PFAS concentration measured for each (non-incubated) plastic type and the average percent PFAS adsorbed for each plastic type in the absence of the associated inorganic and organic matter in relation to the total mass of PFAS spiked into the exposure solution. PFAS concentrations were significantly greater on polyester than polyethylene (*p* < 0.01), but there were no statistically significant differences between polyester and polypropylene or between polypropylene and polyethylene.

All raw data tables are presented in the supplemental section.

## 4. Discussion

Environmental and health concerns over PFAS have increased dramatically in the past few years, although most of that attention has focused on groundwater and soil contamination [29]. In contrast, Remucal [30] measured PFAS concentrations in the open and nearshore Lake Michigan surface waters and found relatively low concentrations of 1.8 to 4.1 ng/L. Although these data are on the low-end of what has previously been reported for PFAS, their proximity to the shore could result in an increased ecosystem stressor [31]. Like a previous study that measured C6 to C10 perfluorocarboxylates and PFOS in Lake Michigan water samples, PFOA, PFHpA, and PFOS were the most commonly found PFAS [30]. PFHxA was not detected in the Muskegon Lake water samples. However, since it was detected on the incubated microplastics, it is likely this PFAS was present but at concentrations below the method detection limit. At the time of analysis for the background lake water samples in this study, the instrument detection limit for PFHxA was a factor of five greater than for other PFASs, such as PFHpA.

After the one month laboratory exposure to PFAS solutions, plastics adsorbed 11% to 36% of the PFAS contained in the exposure solution. A slight trend was observed with regards to the chain length and the amount adsorbed, with the longer chain (PFOA) being adsorbed more than the shorter chain (PFHx). This likely is a function of shorter chains being more water soluble and less adsorbent [32]. In a recent study of adsorption on filter membranes and centrifuge tubes, other researchers found that polypropylene tubes were able to adsorb 32% to 42% of the PFOA in solution that came in contact with this material [33]. Although the exposure time and surface areas were much different than this study, these results are similar.

All plastic types at all locations concentrated PFASs by factors ranging from 24 to 259 times the background lake water concentration. A great deal of variability was observed for PFAS concentrations for duplicate samples of the same type, same location, and same exposure duration. This degree of variability was not observed in the controlled laboratory experiments, analytical duplicate results, or in surrogate recoveries. This suggests that the PFAS distribution is very heterogeneous on the materials. The variability is likely associated with the heterogeneity of the biofilm (plastisphere) colonizing the plastic [34]. The observed variability makes definitive conclusions regarding the effect of plastic type, plastic location, and exposure duration on PFAS adsorption difficult to assess; however, polyethylene deployed in the channel water column drastically increased in PFAS from the 1 month to 3 month period, whereas polypropylene decreased from the 1 month to 3 month time period deployed at the channel bottom.

As part of this field study, adsorption of legacy persistent organic pollutants (POP), such as polycyclic aromatic hydrocarbons (PAH), PCB, and organochlorine pesticides, also were analyzed and the same plastic materials were found to concentrate POP up to 380 times background concentrations, similar in magnitude to what we measured for PFAS [27]. However, in that study, there were clear trends with regards to adsorption on material type (PE > PP > PET), location, and duration. In addition, the variability for samples obtained from the same material, duration, and location was much lower than what was observed for PFAS. The properties of legacy POP and PFAS are considerably different yet the degree of adsorption in the environment was quite similar.

**Figure 7:**
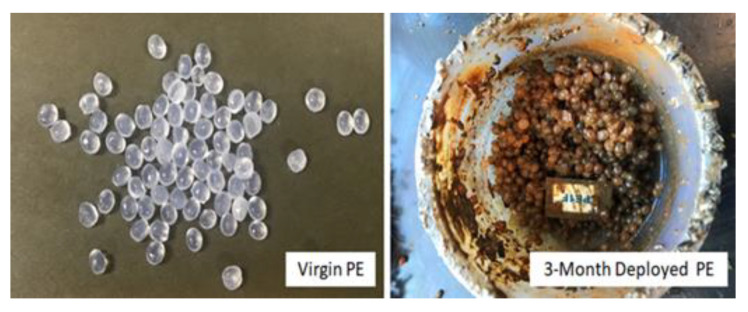
Low Density Polyethylene Before and After Deployment in Muskegon Lake, MI.

The adsorption of PFAS was much greater in the field-incubated plastic than what was observed in the laboratory with plastic and water alone. Figure 7 displays images of polyethylene before and after field deployment (3-month). As shown, the deployed materials when retrieved had a great deal of organic matter and biofilm associated with them, particularly bacteria from the Burkholderiales, Rhodocyclaceae, Comamonadaceae, and Pseudomonadaceae [27]. Previous work has shown that PFASs prefer adsorption to lipids rather than being freely dissolved in water alone [35]. Furthermore, because the biofilm and organic matter accumulation on these materials is heterogenous, this is consistent with the large variability observed in the duplicate PFASs results associated with the same plastic types, locations, and durations reported in the present study. Therefore, the greater degree of PFAS adsorption observed in the field-deployed samples is most likely due to secondary adsorption of these compounds to the plastic-associated organic matter. This is consistent with the findings of Ateia et al. [36], who found that microplastics that were incubated with the natural organic matter had increased uptake of PFOA and PFOS compared to non-incubated microplastics, presumably due to an organic matter formation and/or co-sorption. The role of the biofilm, including the functional roles and adsorptive capacities of its taxonomic composition, is an area in need of additional research [34].

Although microplastics were found to significantly concentrate PFASs from background environmental concentrations, on a per mass basis they are relatively low. In the worst-case scenario found here (polyethylene/channel bottom/3 month duration), the highest concentration of microplastic-associated PFAS was 0.87 ng/g (lowest: 0.052 ng/g). Therefore, for every gram of plastic consumed there exists the potential for an organism to be exposed to an additional ~1 ng of common PFAS. However, it should be noted that several factors could influence the degree of PFAS adsorption. The exposure time of the plastic in Muskegon Lake was relatively short: 1 month and 3 month periods. Modeling studies have suggested that 50% of environmental plastics are 13 years or greater in age. Therefore, the degree of PFAS associated with actual microplastics in the environment may differ from those found in this study. Another factor that can impact the PFAS adsorption is related to the surface area of the microplastics. The size of microplastics in this study (2 mm to 5 mm) is much larger than most microplastics found in the environment. Smaller microplastics would have greater surface area per volume ratios per particle that could potentially provide more active sites of PFASs adsorption. To complicate this issue, PFAS adsorption in the environment appears to be related to secondary adsorption, and increased surface area could potentially facilitate more organic matter adsorption. In addition, over time biofilms can change in composition, which in turn can affect their adsorptive properties. The degree of influence these two parameters may have is unknown. However, it is suspected that they would increase PFASs adsorption, thereby making the results from this study biased low and conservative.

## 5. Conclusions

Three plastic materials (polyethylene, polypropylene, and polyethylene terephthalate) were shown to adsorb PFAS in aqueous environments. Materials deployed in the field (Muskegon Lake) demonstrated a much greater capacity for adsorption than those treated in the laboratory with PFAS and water alone. Concentrations of PFAS associated with plastic materials used in this study were relatively low and of themselves would not likely induce acute adverse effects to organisms exposed to them. However, given the short exposure times of these materials in the environment (3 months maximum) and large particle sizes (2 mm to 4 mm), these results are most likely a conservative estimate for microplastic adsorption of PFAS. These findings also demonstrate the need to consider not only the potential adverse effects of organisms exposed to microplastics alone but also the need to consider the biological and chemical materials associated with plastic materials in the environment.

## Data Availability

Data has been provided in supplementary section of this paper and can be made available on request from the corresponding author.

## Supplementary Materials

The following are available online at https://www.mdpi.com/article/10.3390/toxics9050106/s1, Table S1: Environmental data from the three MP deployment locations at the time of retrieval from Muskegon Lake, Table S2: Final Target Per- and Polyfluoroalkyl Substances (PFAS)—Channel Waters, Table S3: Final Target Per- and Polyfluoroalkyl Substances (PFAS)—Lake Waters, Table S4: Per- and Polyfluoroalkyl Substances (PFAS) Quality Control Summary—Channel and Lake Water Samples, Table S5: Final Target Per- and Polyfluoroalkyl Substances (PFAS)—Virgin Polyethylene, Table S6: Final Target Per- and Polyfluoroalkyl Substances (PFAS)—Virgin Polypropylene, Table S7: Final Target Per- and Polyfluoroalkyl Substances (PFAS)—Virgin Polyester, Table S8: Per- and Polyfluoroalkyl Substances (PFAS) Quality Control Summary—Virgin Plastics, Table S9: Final Target Per- and Polyfluoroalkyl Substances (PFAS)—Polyethylene Deployed for 1 Month—Channel Water, Table S10: Final Target Per- and Polyfluoroalkyl Substances (PFAS)—Polypropylene Deployed for 1 Month—Channel Water, Table S11: Final Target Per- and Polyfluoroalkyl Substances (PFAS)—Polyester Deployed for 1 Month—Channel Water, Table S12: Final Target Per- and Polyfluoroalkyl Substances (PFAS)—Polyethylene Deployed for 1 Month—Channel Bottom, Table S13: Final Target Per- and Polyfluoroalkyl Substances (PFAS)—Polypropylene Deployed for 1 Month—Channel Bottom, Table S14: Final Target Per- and Polyfluoroalkyl Substances (PFAS)—Polyester Deployed for 1 Month—Channel Bottom, Table S15: Final Target Per- and Polyfluoroalkyl Substances (PFAS)—Polyethylene Deployed for 1 Month—Lake Bottom, Table S16: Final Target Per- and Polyfluoroalkyl Substances (PFAS)—Polypropylene Deployed for 1 Month—Lake Bottom, Table S17: Final Target Per- and Polyfluoroalkyl Substances (PFAS)—Polyester Deployed for 1 Month—Lake Bottom, Table S18: Per- and Polyfluoroalkyl Substances (PFAS) Quality Control Summary—1-Month Deployed Plastics, Table S19: Final Target Per- and Polyfluoroalkyl Substances (PFAS)—Polyethylene Deployed for 3 Months—Channel Water, Table S20: Final Target Per- and Polyfluoroalkyl Substances (PFAS)—Polypropylene Deployed for 3 Months—Channel Water, Table S21: Final Target Per- and Polyfluoroalkyl Substances (PFAS)—Polyester Deployed for 3 Months—Channel Water, Table S22: Final Target Per- and Polyfluoroalkyl Substances (PFAS)—Polyethylene Deployed for 3 Months—Channel Bottom, Table S23: Final Target Per- and Polyfluoroalkyl Substances (PFAS)—Polypropylene Deployed for 3 Months—Channel Bottom, Table S24: Final Target Per- and Polyfluoroalkyl Substances (PFAS)—Polyester Deployed for 3 Months—Channel Bottom, Table S25: Final Target Per- and Polyfluoroalkyl Substances (PFAS)—Polyethylene Deployed for 3 Months—Lake Bottom, Table S26: Final Target Per- and Polyfluoroalkyl Substances (PFAS)—Polypropylene Deployed for 3 Months—Lake Bottom, Table S27: Final Target Per- and Polyfluoroalkyl Substances (PFAS)—Polyester Deployed for 3 Months—Lake Bottom, Table S28: Per- and Polyfluoroalkyl Substances (PFAS) Quality Control Summary—3 Month Deployed Plastics
