# Supplementary material for: Perfluoroalkylated Substances (PFAS) Associated with Microplastics in a Lake Environment
Source: Toxics. 2021 May 11;9(5):106. doi: 10.3390/toxics9050106 (PMC8151042; doi:10.3390/toxics9050106)
Supplement: Supplementary file 1 [file toxics-09-00106-s001.zip › toxics-1184314-supplementary.pdf]

toxics

MDPI

Supplemental Section – Raw data and Quality Control reports

Table S1 : Final Target Per- and polyfluoroalkyl Substances (PFAS) - Channel Waters

Matrix: Aqueous - Collected 5/7/2019

Date Extracted: 05/09/2019 | Sample Prep: EPA Method 537

Data Analyzed: 05/15/2019 & 08/05/19 | Instrument: Waters Quattro Micro LC-Tandem Mass Spectrometer

Identification	Channel Water #1	Channel Water #2	Average	%RPD
Units	µg/L	µg/L	µg/L	
**Native PFAS**				
Perfluorobutanesulfonic acid	0.00072	0.00098	0.00085	31%
Perfluorohexanoic acid	< 0.0005	< 0.0005	< 0.0005	NA
Perfluoroheptanoic acid	0.00051	0.00037	0.00044	31%
Perfluorooctanoic acid	0.0010	0.0013	0.0011	25%
Perfluorononanoic acid	< 0.0005	< 0.0005	< 0.0005	NA
Perfluorodecanoic acid	< 0.0001	< 0.0001	< 0.0001	NA
Perfluorooctanesulfonic acid	0.00040	0.00044	0.00042	9.4%
Sum Target PFAS	0.0026	0.0031	0.0028	16%

Surrogate PFAS	%Recovery	%Recovery	Average	%RPD
Perfluorobutanesulfonic acid	71%	97%	84%	31%
Perfluorohexanoic acid	95%	87%	91%	9.4%
Perfluoroheptanoic acid	59%	53%	56%	9.9%
Perfluorooctanoic acid	68%	60%	64%	13%
Perfluorononanoic acid	68%	53%	61%	25%
Perfluorodecanoic acid	46%	30%	38%	44%
Perfluorooctanesulfonic acid	51%	63%	57%	22%
Average Surrogate Recovery	65%	63%	64%	22%

NA - Not Applicable

Table S2 : Final Target Per- and polyfluoroalkyl Substances (PFAS) - Lake Waters

Matrix: Aqueous - Collected 5/7/2019
Date Extracted: 05/09/2019 — **Sample Prep: EPA Method 537**
Data Analyzed: 05/15/2019 & 08/05/19 — **Instrument: Waters Quattro Micro LC-Tandem Mass Spectrometer**

Identification	Lake Water #1	Lake Water #2	Average	%RPD
Units	µg/L	µg/L	µg/L	
**Native PFAS**				
Perfluorobutanesulfonic acid	0.00093	0.00069	0.00081	30%
Perfluorohexanoic acid	< 0.0005	< 0.0005	< 0.0005	NA
Perfluoroheptanoic acid	0.00035	0.00039	0.00037	12%
Perfluorooctanoic acid	0.0021	0.0017	0.0019	18%
Perfluorononanoic acid	< 0.0005	< 0.0005	< 0.0005	NA
Perfluorodecanoic acid	< 0.0001	< 0.0001	< 0.0001	NA
Perfluorooctanesulfonic acid	< 0.0001	0.00040	0.00040	NA
Sum Target PFAS	0.0034	0.0032	0.0033	4.2%
**Surrogate PFAS**	**%Recovery**	**%Recovery**	**Average**	**%RPD**
Perfluorobutanesulfonic acid	100%	88%	94%	13%
Perfluorohexanoic acid	75%	66%	70%	14%
Perfluoroheptanoic acid	58%	77%	68%	28%
Perfluorooctanoic acid	60%	85%	72%	34%
Perfluorononanoic acid	41%	81%	61%	65%
Perfluorodecanoic acid	17%	51%	34%	99%
Perfluorooctanesulfonic acid	36%	50%	43%	34%
Average Surrogate Recovery	55%	71%	63%	41%

NA - Not Applicable

Table S3: Per- and polyfluoroalkyl Substances (PFAS) Quality Control Summary - Channel and Lake Water Samples

Identification			-	Lake Water #2	Channel Water #2	Channel Water
	µg/L	µg/L	%Recovery	%Recovery	%RPD	%Recovery
	Reagent Blank	Trip Blank	Reagent Blank Spike	Analytical Spike	Analytical Sample Duplicate	Sample Matrix Spike
Perfluorobutanesulfonic acid	< 0.0005	< 0.0005	104%	128%	23%	51%
Perfluorohexanoic acid	< 0.0005	< 0.0005	111%	125%	NC1	60%
Perfluoroheptanoic acid	< 0.0001	< 0.0001	105%	121%	4%	71%
Perfluorooctanoic acid	< 0.0005	< 0.0005	116%	90%	15%	89%
Perfluorononanoic acid	< 0.0005	< 0.0005	91%	129%	NC1	80%
Perfluorodecanoic acid	< 0.0001	< 0.0001	83%	123%	NC1	42%
Perfluorooctanesulfonic acid	< 0.0001	< 0.0001	114%	130%	24%	55%
**Average Recovery & RPD**	NA	NA	103%	121%	17%	64%
**Standard Deviation**	NA	NA	12%	14%	56%	17%

NA - Not Applicable

NC1 - Not calculated, less than detection limit.

Table S4 : Final Target Per- and polyfluoroalkyl Substances (PFAS) - Virgin Polyethylene

Date Extracted: 03/19/2019
Data Analyzed: 07/15/2019

Matrix: Polyethylene (PE)
Sample Prep: Solid-Liquid Extraction
Instrument: Waters Quattro Micro LC-Tandem Mass Spectrometer

Identification	Virgin PE #1	Virgin PE #2	Average	%RPD
Units	ng/g	ng/g	ng/g	
**Native PFAS**				
Perfluorobutanesulfonic acid	< 0.05	< 0.05	< 0.05	NA
Perfluorohexanoic acid	< 0.05	< 0.05	< 0.05	NA
Perfluoroheptanoic acid	< 0.05	< 0.05	< 0.05	NA
Perfluorooctanoic acid	< 0.05	< 0.05	< 0.05	NA
Perfluorononanoic acid	< 0.05	< 0.05	< 0.05	NA
Perfluorodecanoic acid	< 0.05	< 0.05	< 0.05	NA
Perfluorooctanesulfonic acid	< 0.05	< 0.05	< 0.05	NA
Sum Target PFAS	Non-Detect	Non-Detect	Non-Detect	NA
**Surrogate PFAS**	%Recovery	%Recovery	Average	%RPD
Perfluorobutanesulfonic acid	91%	73%	82%	22%
Perfluorohexanoic acid	83%	91%	87%	9.2%
Perfluoroheptanoic acid	88%	96%	92%	8.7%
Perfluorooctanoic acid	94%	90%	92%	4.9%
Perfluorononanoic acid	79%	84%	81%	6.2%
Perfluorodecanoic acid	51%	51%	51%	0.0%
Perfluorooctanesulfonic acid	63%	58%	60%	8.3%
Average Surrogate Recovery	78%	77%	78%	8.5%

NA - Not Applicable

Table S5 : Final Target Per- and polyfluoroalkyl Substances (PFAS) - Virgin Polyproylene

Matrix: Polyproylene (PP)
Date Extracted: 03/12/2019 — Sample Prep: Solid-Liquid Extraction
Data Analyzed: 07/15/2019 — Instrument: Waters Quattro Micro LC-Tandem Mass Spectrometer

Identification	Virgin PP #1	Virgin PP #2	Average	%RPD
Units	ng/g	ng/g	ng/g	
**Native PFAS**				
Perfluorobutanesulfonic acid	< 0.05	< 0.05	< 0.05	NA
Perfluorohexanoic acid	< 0.05	< 0.05	< 0.05	NA
Perfluoroheptanoic acid	< 0.05	< 0.05	< 0.05	NA
Perfluorooctanoic acid	< 0.05	< 0.05	< 0.05	NA
Perfluorononanoic acid	< 0.05	< 0.05	< 0.05	NA
Perfluorodecanoic acid	< 0.05	< 0.05	< 0.05	NA
Perfluorooctanesulfonic acid	< 0.05	< 0.05	< 0.05	NA
Sum Target PFAS	Non-Detect	Non-Detect	Non-Detect	NA
**Surrogate PFAS**	**%Recovery**	**%Recovery**	**Average**	**%RPD**
Perfluorobutanesulfonic acid	80%	92%	86%	13%
Perfluorohexanoic acid	83%	79%	81%	4.3%
Perfluoroheptanoic acid	88%	81%	84%	8.9%
Perfluorooctanoic acid	87%	88%	87%	0.57%
Perfluorononanoic acid	93%	82%	87%	13%
Perfluorodecanoic acid	79%	69%	74%	14%
Perfluorooctanesulfonic acid	75%	63%	69%	18%
Average Surrogate Recovery	83%	79%	81%	10%

NA - Not Applicable

Table S6 : Final Target Per- and polyfluoroalkyl Substances (PFAS) - Virgin Polyester

IISG PFAS- Microplastics Study	Matrix: Polyproylene (PET)
Date Extracted: 03/12/219	Sample Prep: Solid-Liquid Extraction
Data Analyzed: 07/15/2019	Instrument: Waters Quattro Micro LC-Tandem Mass Spectrometer

Identification	Virgin PET #1	Virgin PET #2	Average	%RPD
Units	ng/g	ng/g	ng/g	
**Native PFAS**				
Perfluorobutanesulfonic acid	< 0.05	< 0.05	< 0.05	NA
Perfluorohexanoic acid	< 0.05	< 0.05	< 0.05	NA
Perfluoroheptanoic acid	< 0.05	< 0.05	< 0.05	NA
Perfluorooctanoic acid	< 0.05	< 0.05	< 0.05	NA
Perfluorononanoic acid	< 0.05	< 0.05	< 0.05	NA
Perfluorodecanoic acid	< 0.05	< 0.05	< 0.05	NA
Perfluorooctanesulfonic acid	< 0.05	< 0.05	< 0.05	NA
Sum Target PFAS	Non-Detect	Non-Detect	Non-Detect	NA
**Surrogate PFAS**	**%Recovery**	**%Recovery**	**Average**	**%RPD**
Perfluorobutanesulfonic acid	83%	102%	92%	21%
Perfluorohexanoic acid	88%	60%	74%	37%
Perfluoroheptanoic acid	92%	67%	79%	31%
Perfluorooctanoic acid	94%	67%	80%	34%
Perfluorononanoic acid	98%	77%	87%	25%
Perfluorodecanoic acid	88%	66%	77%	28%
Perfluorooctanesulfonic acid	81%	93%	87%	14%
Average Surrogate Recovery	89%	76%	82%	27%

NA - Not Applicable

Table S7: Per- and polyfluoroalkyl Substances (PFAS) Quality Control Summary - Virgin Plastics

Identification		-	Virgin Polyethylene	Virgin Polyester	Virgin Polyester
	ng/g	%Recovery	%Recovery	%RPD	%Recovery
	Reagent Blank	Reagent Blank Spike	Analytical Spike	Analytical Sample Duplicate	Sample Matrix Spike
Perfluorobutanesulfonic acid	< 0.05	93%	95%	NC1	104%
Perfluorohexanoic acid	< 0.05	62%	84%	NC1	52%
Perfluoroheptanoic acid	< 0.05	89%	78%	NC1	85%
Perfluorooctanoic acid	< 0.05	91%	77%	NC1	85%
Perfluorononanoic acid	< 0.05	68%	73%	NC1	83%
Perfluorodecanoic acid	< 0.05	42%	71%	NC1	79%
Perfluorooctanesulfonic acid	< 0.05	66%	70%	NC1	92%
**Average Recovery & RPD**	NA	73%	78%	NC1	83%
**Standard Deviation**	NA	19%	8.6%	NA	16%

NA - Not Applicable

NC1 - Not calculated, less than detection limit.

Table S8 : Final Target Per- and polyfluoroalkyl Substances (PFAS) - Polyethylene Deployed for 1 Month - Channel Water

Date Extracted: 5/6/2019

Data Analyzed: 8/29/2019

Matrix: Polyethylene (PE)

Sample Prep: Solid-Liquid Extraction

Instrument: Waters Quattro Micro LC-Tandem Mass Spectrometer

Identification	PE - Channel Water #1	PE- Channel Water #2	Average	%RPD
Units	ng/g	ng/g	ng/g	
**Native PFAS**				
Perfluorobutanesulfonic acid	< 0.05	< 0.05	< 0.05	NA
Perfluorohexanoic acid	0.051	0.060	0.055	17%
Perfluoroheptanoic acid	0.074	0.071	0.074	5.1%
Perfluorooctanoic acid	0.094	0.12	0.11	21%
Perfluorononanoic acid	< 0.05	< 0.05	< 0.05	NA
Perfluorodecanoic acid	< 0.05	< 0.05	< 0.05	NA
Perfluorooctanesulfonic acid	< 0.05	< 0.05	< 0.05	NA
Sum Target PFAS	0.22	0.25	0.23	12%
**Surrogate PFAS**	**%Recovery**	**%Recovery**	**Average**	**%RPD**
Perfluorobutanesulfonic acid	109%	99%	104%	10%
Perfluorohexanoic acid	118%	100%	109%	17%
Perfluoroheptanoic acid	94%	99%	97%	5.2%
Perfluorooctanoic acid	106%	77%	92%	32%
Perfluorononanoic acid	67%	67%	67%	0.0%
Perfluorodecanoic acid	41%	26%	34%	45%
Perfluorooctanesulfonic acid	68%	57%	63%	18%
Average Surrogate Recovery	86%	75%	81%	18%

NA - Not Applicable

Table S9 : Final Target Per- and polyfluoroalkyl Substances (PFAS) - Polypropylene Deployed for 1 Month - Channel Water

Matrix: Polypropylene (PP)

Date Extracted: 5/6/2019 **Sample Prep: Solid-Liquid Extraction**

Data Analyzed: 8/29/19 **Instrument: Waters Quattro Micro LC-Tandem Mass Spectrometer**

Identification	PP - Channel Water #1	PP- Channel Water #2	Average	%RPD
Units	ng/g	ng/g	ng/g	
**Native PFAS**				
Perfluorobutanesulfonic acid	< 0.05	< 0.05	< 0.05	NA
Perfluorohexanoic acid	0.092	< 0.05	0.092	NA
Perfluoroheptanoic acid	0.20	0.095	0.20	52%
Perfluorooctanoic acid	0.13	0.10	0.12	24%
Perfluorononanoic acid	< 0.05	< 0.05	< 0.05	NA
Perfluorodecanoic acid	< 0.05	< 0.05	< 0.05	NA
Perfluorooctanesulfonic acid	< 0.05	< 0.05	< 0.05	NA
Sum Target PFAS	0.42	0.20	0.31	72%
**Surrogate PFAS**	**%Recovery**	**%Recovery**	**Average**	**%RPD**
Perfluorobutanesulfonic acid	68%	78%	73%	14%
Perfluorohexanoic acid	65%	84%	75%	26%
Perfluoroheptanoic acid	71%	84%	78%	17%
Perfluorooctanoic acid	62%	89%	76%	36%
Perfluorononanoic acid	67%	50%	59%	29%
Perfluorodecanoic acid	30%	31%	31%	3.3%
Perfluorooctanesulfonic acid	43%	39%	41%	10%
Average Surrogate Recovery	58%	65%	62%	19%

NA - Not Applicable

Table S10 : Final Target Per- and polyfluoroalkyl Substances (PFAS) - Polyester Deployed for 1 Month - Channel Water

Matrix: Polyester (PET)

Date Extracted: 5/6/2019 — Sample Prep: Solid-Liquid Extraction

Data Analyzed: 8/29/2019 — Instrument: Waters Quattro Micro LC-Tandem Mass Spectrometer

Identification	PET - Channel Water #1	PET- Channel Water #2	Average	%RPD
Units	ng/g	ng/g	ng/g	
**Native PFAS**				
Perfluorobutanesulfonic acid	< 0.05	< 0.05	< 0.05	NA
Perfluorohexanoic acid	0.099	0.079	0.089	22%
Perfluoroheptanoic acid	0.089	0.060	0.089	33%
Perfluorooctanoic acid	0.10	0.075	0.088	30%
Perfluorononanoic acid	< 0.05	< 0.05	< 0.05	NA
Perfluorodecanoic acid	< 0.05	< 0.05	< 0.05	NA
Perfluorooctanesulfonic acid	< 0.05	< 0.05	< 0.05	NA
Sum Target PFAS	0.29	0.15	0.22	61%
**Surrogate PFAS**	%Recovery	%Recovery	Average	%RPD
Perfluorobutanesulfonic acid	78%	66%	72%	17%
Perfluorohexanoic acid	71%	63%	67%	12%
Perfluoroheptanoic acid	79%	67%	73%	16%
Perfluorooctanoic acid	79%	67%	73%	16%
Perfluorononanoic acid	53%	53%	53%	0.0%
Perfluorodecanoic acid	24%	25%	25%	4.1%
Perfluorooctanesulfonic acid	53%	46%	50%	14%
Average Surrogate Recovery	62%	55%	59%	11%

NA - Not Applicable

Table S11 : Final Target Per- and polyfluoroalkyl Substances (PFAS) - Polyethylene Deployed for 1 Month - Channel Bottom

Matrix: Polyethylene (PE)

Date Extracted: 5/6/2019 — **Sample Prep: Solid-Liquid Extraction**

Data Analyzed: 8/29/2019 & 9/23/2019 — **Instrument: Waters Quattro Micro LC-Tandem Mass Spectrometer**

Identification	PE - Channel Bottom #1	PE - Channel Bottom #2	Average	%RPD
Units	ng/g	ng/g	ng/g	
**Native PFAS**				
Perfluorobutanesulfonic acid	< 0.05	< 0.05	< 0.05	NA
Perfluorohexanoic acid	< 0.05	0.065	0.065	NA
Perfluoroheptanoic acid	< 0.05	< 0.05	< 0.05	NA
Perfluorooctanoic acid	0.21	0.11	0.16	61%
Perfluorononanoic acid	< 0.05	< 0.05	< 0.05	NA
Perfluorodecanoic acid	< 0.05	< 0.05	< 0.05	NA
Perfluorooctanesulfonic acid	< 0.05	< 0.05	< 0.05	NA
Sum Target PFAS	0.21	0.18	0.19	18%
**Surrogate PFAS**	**%Recovery**	**%Recovery**	**Average**	**%RPD**
Perfluorobutanesulfonic acid	81%	82%	82%	1.2%
Perfluorohexanoic acid	81%	93%	87%	14%
Perfluoroheptanoic acid	78%	67%	73%	15%
Perfluorooctanoic acid	62%	54%	58%	14%
Perfluorononanoic acid	28%	40%	34%	37%
Perfluorodecanoic acid	11%	14%	12%	29%
Perfluorooctanesulfonic acid	37%	58%	48%	44%
Average Surrogate Recovery	54%	58%	56%	22%

NA - Not Applicable

Table S12 : Final Target Per- and polyfluoroalkyl Substances (PFAS) - Polypropylene Deployed for 1 Month - Channel Bottom

Matrix: Polypropylene (PP)
Date Extracted: 5/6/2019 — Sample Prep: Solid-Liquid Extraction
Data Analyzed: 8/29/2019 — Instrument: Waters Quattro Micro LC-Tandem Mass Spectrometer

Identification	PP - Channel Bottom #1	PP - Channel Bottom #2	Average	%RPD
Units	ng/g	ng/g	ng/g	
**Native PFAS**				
Perfluorobutanesulfonic acid	< 0.05	< 0.05	< 0.05	NA
Perfluorohexanoic acid	< 0.05	0.0722	0.072	NA
Perfluoroheptanoic acid	0.095	0.0583	0.077	48%
Perfluorooctanoic acid	0.10	0.1000	0.10	1.1%
Perfluorononanoic acid	< 0.05	< 0.05	< 0.05	NA
Perfluorodecanoic acid	< 0.05	< 0.05	< 0.05	NA
Perfluorooctanesulfonic acid	< 0.05	< 0.05	< 0.05	NA
Sum Target PFAS	0.20	0.23	0.21	16%
**Surrogate PFAS**	%Recovery	%Recovery	Average	%RPD
Perfluorobutanesulfonic acid	78%	116%	97%	39%
Perfluorohexanoic acid	84%	97%	91%	14%
Perfluoroheptanoic acid	84%	103%	94%	20%
Perfluorooctanoic acid	89%	100%	95%	12%
Perfluorononanoic acid	50%	46%	48%	8.3%
Perfluorodecanoic acid	31%	21%	26%	38%
Perfluorooctanesulfonic acid	39%	47%	43%	19%
Average Surrogate Recovery	65%	76%	70%	22%

NA - Not Applicable

Table S13 : Final Target Per- and polyfluoroalkyl Substances (PFAS) - Polyester Deployed for 1 Month - Channel Bottom

Matrix: Polyester (PET)

Date Extracted: 5/6/2019 — Sample Prep: Solid-Liquid Extraction

Data Analyzed: 8/29/2019 — Instrument: Waters Quattro Micro LC-Tandem Mass Spectrometer

Identification	PET - Channel Bottom #1	PET - Channel Bottom #2	Average	%RPD
Units	ng/g	ng/g	ng/g	
**Native PFAS**				
Perfluorobutanesulfonic acid	< 0.05	< 0.05	< 0.05	NA
Perfluorohexanoic acid	0.11	< 0.05	0.11	NA
Perfluoroheptanoic acid	0.10	0.10	0.10	0.0%
Perfluorooctanoic acid	0.10	0.14	0.12	29%
Perfluorononanoic acid	< 0.05	< 0.05	< 0.05	NA
Perfluorodecanoic acid	< 0.05	< 0.05	< 0.05	NA
Perfluorooctanesulfonic acid	< 0.05	< 0.05	< 0.05	NA
Sum Target PFAS	0.31	0.24	0.27	26%
**Surrogate PFAS**	**%Recovery**	**%Recovery**	**Average**	**%RPD**
Perfluorobutanesulfonic acid	63%	54%	59%	15%
Perfluorohexanoic acid	57%	54%	56%	5.4%
Perfluoroheptanoic acid	70%	60%	65%	15%
Perfluorooctanoic acid	78%	58%	68%	29%
Perfluorononanoic acid	35%	43%	39%	21%
Perfluorodecanoic acid	13%	18%	16%	32%
Perfluorooctanesulfonic acid	42%	39%	41%	7.4%
Average Surrogate Recovery	51%	47%	49%	18%

NA - Not Applicable

Table S14 : Final Target Per- and polyfluoroalkyl Substances (PFAS) - Polyethylene Deployed for 1 Month - Lake Bottom

Matrix: Polyethylene (PE)

Date Extracted: 04/30/2019 | Sample Prep: Solid-Liquid Extraction

Data Analyzed: 08/29/2019 | Instrument: Waters Quattro Micro LC-Tandem Mass Spectrometer

Identification	PE - Lake Bottom #1	PE - Lake Bottom #2	Average	%RPD
Units	ng/g	ng/g	ng/g	
**Native PFAS**				
Perfluorobutanesulfonic acid	< 0.05	< 0.05	< 0.05	NA
Perfluorohexanoic acid	0.081	< 0.05	0.081	NA
Perfluoroheptanoic acid	0.063	0.059	0.063	6.4%
Perfluorooctanoic acid	0.13	0.29	0.21	75%
Perfluorononanoic acid	< 0.05	< 0.05	< 0.05	NA
Perfluorodecanoic acid	< 0.05	< 0.05	< 0.05	NA
Perfluorooctanesulfonic acid	< 0.05	< 0.05	< 0.05	NA
Sum Target PFAS	0.28	0.35	0.32	24%
**Surrogate PFAS**	%Recovery	%Recovery	Average	%RPD
Perfluorobutanesulfonic acid	73%	66%	69%	11%
Perfluorohexanoic acid	74%	69%	71%	7.7%
Perfluoroheptanoic acid	79%	68%	73%	16%
Perfluorooctanoic acid	105%	68%	87%	43%
Perfluorononanoic acid	60%	27%	43%	77%
Perfluorodecanoic acid	30%	13%	22%	79%
Perfluorooctanesulfonic acid	69%	36%	53%	63%
Average Surrogate Recovery	70%	49%	60%	42%

NA - Not Applicable

Table S15 : Final Target Per- and polyfluoroalkyl Substances (PFAS) - Polypropylene Deployed for 1 Month - Lake Bottom

Matrix: Polypropylene (PP)
Date Extracted: 04/30/2019 Sample Prep: Solid-Liquid Extraction
Data Analyzed: 08/29/2019 Instrument: Waters Quattro Micro LC-Tandem Mass Spectrometer

Identification	PP - Lake Bottom #1	PP - Lake Bottom #2	Average	%RPD
Units	ng/g	ng/g	ng/g	
**Native PFAS**				
Perfluorobutanesulfonic acid	< 0.05	< 0.05	< 0.05	NA
Perfluorohexanoic acid	0.085	< 0.05	0.085	NA
Perfluoroheptanoic acid	< 0.05	< 0.05	< 0.05	NA
Perfluorooctanoic acid	0.11	0.15	0.13	31%
Perfluorononanoic acid	< 0.05	< 0.05	< 0.05	NA
Perfluorodecanoic acid	< 0.05	< 0.05	< 0.05	NA
Perfluorooctanesulfonic acid	< 0.05	< 0.05	< 0.05	NA
Sum Target PFAS	0.19	0.15	0.17	26%
**Surrogate PFAS**	%Recovery	%Recovery	Average	%RPD
Perfluorobutanesulfonic acid	71%	75%	73%	5.5%
Perfluorohexanoic acid	71%	69%	70%	2.9%
Perfluoroheptanoic acid	74%	70%	72%	5.6%
Perfluorooctanoic acid	60%	65%	63%	8.0%
Perfluorononanoic acid	45%	53%	49%	16%
Perfluorodecanoic acid	14%	17%	16%	19%
Perfluorooctanesulfonic acid	53%	60%	57%	12%
Average Surrogate Recovery	55%	58%	57%	10%

NA - Not Applicable

Table S16 : Final Target Per- and polyfluoroalkyl Substances (PFAS) - Polyester Deployed for 1 Month - Lake Bottom

Matrix: Polyester (PET)

Date Extracted: 04/30/2019 — Sample Prep: Solid-Liquid Extraction

Data Analyzed: 08/29/2019 — Instrument: Waters Quattro Micro LC-Tandem Mass Spectrometer

Identification	PET - Lake Bottom #1	PET - Lake Bottom #2	Average	%RPD
Units	ng/g	ng/g	ng/g	
**Native PFAS**				
Perfluorobutanesulfonic acid	< 0.05	< 0.05	< 0.05	NA
Perfluorohexanoic acid	< 0.05	0.093	0.093	NA
Perfluoroheptanoic acid	0.12	0.18	0.12	48%
Perfluorooctanoic acid	0.11	0.18	0.15	43%
Perfluorononanoic acid	< 0.05	< 0.05	< 0.05	NA
Perfluorodecanoic acid	< 0.05	< 0.05	< 0.05	NA
Perfluorooctanesulfonic acid	< 0.05	< 0.05	< 0.05	NA
Sum Target PFAS	0.23	0.27	0.24	14%
**Surrogate PFAS**	%Recovery	%Recovery	Average	%RPD
Perfluorobutanesulfonic acid	77%	65%	71%	17%
Perfluorohexanoic acid	67%	53%	60%	23%
Perfluoroheptanoic acid	67%	54%	61%	21%
Perfluorooctanoic acid	79%	51%	65%	43%
Perfluorononanoic acid	40%	34%	37%	16%
Perfluorodecanoic acid	26%	21%	24%	21%
Perfluorooctanesulfonic acid	46%	48%	47%	4.3%
Average Surrogate Recovery	57%	47%	52%	21%

NA - Not Applicable

Table S17: Per- and polyfluoroalkyl Substances (PFAS) Quality Control Summary - 1-Month Deployed Plastics

Identification		-	PE - Lake Bottom	PE - Channel Bottom	PP - Lake Bottom
	ng/g	%Recovery	%Recovery	%RPD	%Recovery
	Reagent Blank	Reagent Blank Spike	Analytical Spike	Analytical Sample Duplicate	Sample Matrix Spike
Perfluorobutanesulfonic acid	< 0.05	88%	89%	NC1	108%
Perfluorohexanoic acid	< 0.05	80%	85%	NC1	58%
Perfluoroheptanoic acid	< 0.05	119%	88%	NC1	136%
Perfluorooctanoic acid	< 0.05	69%	73%	26%	53%
Perfluorononanoic acid	< 0.05	107%	89%	NC1	127%
Perfluorodecanoic acid	< 0.05	138%	80%	NC1	80%
Perfluorooctanesulfonic acid	< 0.05	86%	85%	NC1	140%
**Average Recovery & RPD**	NA	98%	84%	26%	100%
**Standard Deviation**	NA	24%	5.8%	NA	37%

NA - Not Applicable

NC1 - Not calculated, less than detection limit.

Table S18 : Final Target Per- and polyfluoroalkyl Substances (PFAS) - Polyethylene Deployed for 3 Months - Channel Water

Matrix: Polyethylene (PE)
Date Extracted: 3/20/2019 & 5/6/2019 — Sample Prep: Solid-Liquid Extraction
Data Analyzed: 07/01/2019 & 08/12/19 — Instrument: Waters Quattro Micro LC-Tandem Mass Spectrometer

Identification	PE - Channel Water #1	PE- Channel Water #2	Average	%RPD
Units	ng/g	ng/g	ng/g	
**Native PFAS**				
Perfluorobutanesulfonic acid	< 0.05	< 0.05	< 0.05	NA
Perfluorohexanoic acid	0.11	0.11	0.11	4.3%
Perfluoroheptanoic acid	0.20	< 0.05	0.20	NA
Perfluorooctanoic acid	0.55	0.39	0.47	34%
Perfluorononanoic acid	< 0.05	< 0.05	< 0.05	NA
Perfluorodecanoic acid	< 0.05	< 0.05	< 0.05	NA
Perfluorooctanesulfonic acid	< 0.05	0.10	0.10	NA
Sum Target PFAS	0.87	0.60	0.73	37%
**Surrogate PFAS**	**%Recovery**	**%Recovery**	**Average**	**%RPD**
Perfluorobutanesulfonic acid	94%	66%	80%	35%
Perfluorohexanoic acid	88%	64%	76%	32%
Perfluoroheptanoic acid	84%	64%	74%	28%
Perfluorooctanoic acid	68%	56%	62%	20%
Perfluorononanoic acid	27%	34%	30%	23%
Perfluorodecanoic acid	6.5%	12%	9.0%	56%
Perfluorooctanesulfonic acid	41%	42%	41%	2%
Average Surrogate Recovery	58%	48%	53%	28%

NA - Not Applicable

Table S19 : Final Target Per- and polyfluoroalkyl Substances (PFAS) - Polypropylene Deployed for 3 Months - Channel Water

Matrix: Polypropylene (PP)

Date Extracted: 3/20/2019 & 5/6/2019 | Sample Prep: Solid-Liquid Extraction

Data Analyzed: 07/01/2019 & 08/12/19 | Instrument: Waters Quattro Micro LC-Tandem Mass Spectrometer

Identification	PP - Channel Water #1	PP- Channel Water #2	Average	%RPD
Units	ng/g	ng/g	ng/g	
**Native PFAS**				
Perfluorobutanesulfonic acid	< 0.05	< 0.05	< 0.05	NA
Perfluorohexanoic acid	0.054	0.058	0.056	6.5%
Perfluoroheptanoic acid	0.051	< 0.05	0.051	NA
Perfluorooctanoic acid	0.081	0.086	0.083	6.7%
Perfluorononanoic acid	< 0.05	< 0.05	< 0.05	NA
Perfluorodecanoic acid	< 0.05	< 0.05	< 0.05	NA
Perfluorooctanesulfonic acid	< 0.05	< 0.05	< 0.05	NA
Sum Target PFAS	0.19	0.14	0.17	25%
**Surrogate PFAS**	**%Recovery**	**%Recovery**	**Average**	**%RPD**
Perfluorobutanesulfonic acid	106%	92%	99%	15%
Perfluorohexanoic acid	73%	68%	70%	7.8%
Perfluoroheptanoic acid	58%	56%	57%	2.6%
Perfluorooctanoic acid	54%	46%	50%	17%
Perfluorononanoic acid	68%	72%	70%	5.7%
Perfluorodecanoic acid	10%	13%	11%	27%
Perfluorooctanesulfonic acid	67%	69%	68%	3.0%
Average Surrogate Recovery	62%	59%	61%	11%

NA - Not Applicable

Table S20: Final Target Per- and polyfluoroalkyl Substances (PFAS) - Polyester Deployed for 3 Months - Channel Water

Matrix: Polyester (PET)

Date Extracted: 3/20/2019 & 5/6/2019 | Sample Prep: Solid-Liquid Extraction

Data Analyzed: 07/01/2019 & 08/12/19 | Instrument: Waters Quattro Micro LC-Tandem Mass Spectrometer

Identification	PET - Channel Water #1	PET- Channel Water #2	Average	%RPD
Units	ng/g	ng/g	ng/g	
**Native PFAS**				
Perfluorobutanesulfonic acid	< 0.05	< 0.05	< 0.05	NA
Perfluorohexanoic acid	< 0.05	< 0.05	< 0.05	NA
Perfluoroheptanoic acid	< 0.05	0.059	< 0.05	NA
Perfluorooctanoic acid	0.062	0.20	0.13	106%
Perfluorononanoic acid	< 0.05	< 0.05	< 0.05	NA
Perfluorodecanoic acid	< 0.05	< 0.05	< 0.05	NA
Perfluorooctanesulfonic acid	< 0.05	< 0.05	< 0.05	NA
Sum Target PFAS	0.062	0.26	0.16	123%
**Surrogate PFAS**	%Recovery	%Recovery	Average	%RPD
Perfluorobutanesulfonic acid	82%	128%	105%	44%
Perfluorohexanoic acid	47%	60%	53%	25%
Perfluoroheptanoic acid	37%	50%	43%	31%
Perfluorooctanoic acid	30%	35%	32%	14%
Perfluorononanoic acid	25%	28%	26%	13%
Perfluorodecanoic acid	11%	5.5%	8.0%	63%
Perfluorooctanesulfonic acid	27%	28%	27%	1.8%
Average Surrogate Recovery	37%	48%	42%	28%

NA - Not Applicable

Table S21: Final Target Per- and polyfluoroalkyl Substances (PFAS) - Polyethylene Deployed for 3 Months - Channel Bottom

Matrix: Polyethylene (PE)
Date Extracted: 3/20/2019 & 5/6/2019
Sample Prep: Solid-Liquid Extraction
Data Analyzed: 07/01/2019 & 08/12/19
Instrument: Waters Quattro Micro LC-Tandem Mass Spectrometer

Identification	PE - Channel Bottom #1	PE - Channel Bottom #2	Average	%RPD
Units	ng/g	ng/g	ng/g	
**Native PFAS**				
Perfluorobutanesulfonic acid	< 0.05	< 0.05	< 0.05	NA
Perfluorohexanoic acid	0.10	0.062	0.081	48%
Perfluoroheptanoic acid	0.066	0.051	0.066	23%
Perfluorooctanoic acid	0.27	0.098	0.18	93%
Perfluorononanoic acid	< 0.05	< 0.05	< 0.05	NA
Perfluorodecanoic acid	< 0.05	< 0.05	< 0.05	NA
Perfluorooctanesulfonic acid	< 0.05	< 0.05	< 0.05	NA
Sum Target PFAS	0.43	0.21	0.32	69%
**Surrogate PFAS**	%Recovery	%Recovery	Average	%RPD
Perfluorobutanesulfonic acid	122%	129%	126%	5.6%
Perfluorohexanoic acid	88%	86%	87%	1.7%
Perfluoroheptanoic acid	81%	104%	92%	25%
Perfluorooctanoic acid	63%	90%	77%	35%
Perfluorononanoic acid	34%	43%	38%	22%
Perfluorodecanoic acid	8.5%	11%	9.5%	21%
Perfluorooctanesulfonic acid	25%	27%	26%	7.8%
Average Surrogate Recovery	60%	70%	65%	17%

NA - Not Applicable

Table S22 : Final Target Per- and polyfluoroalkyl Substances (PFAS) - Polypropylene Deployed for 3 Months - Channel Bottom

Matrix: Polypropylene (PP)

Date Extracted: 3/20/2019 & 5/6/2019 — Sample Prep: Solid-Liquid Extraction

Data Analyzed: 9/23/219 — Instrument: Waters Quattro Micro LC-Tandem Mass Spectrometer

Identification	PP - Channel Bottom #1	PP - Channel Bottom #2	Average	%RPD
Units	ng/g	ng/g	ng/g	
**Native PFAS**				
Perfluorobutanesulfonic acid	< 0.05	< 0.05	< 0.05	NA
Perfluorohexanoic acid	< 0.05	< 0.05	< 0.05	NA
Perfluoroheptanoic acid	< 0.05	< 0.05	< 0.05	NA
Perfluorooctanoic acid	0.083	0.052	0.067	47%
Perfluorononanoic acid	< 0.05	< 0.05	< 0.05	NA
Perfluorodecanoic acid	< 0.05	< 0.05	< 0.05	NA
Perfluorooctanesulfonic acid	< 0.05	< 0.05	< 0.05	NA
Sum Target PFAS	0.083	0.052	0.067	47%
**Surrogate PFAS**	**%Recovery**	**%Recovery**	**Average**	**%RPD**
Perfluorobutanesulfonic acid	69%	53%	61%	26%
Perfluorohexanoic acid	60%	55%	57%	10%
Perfluoroheptanoic acid	67%	52%	60%	25%
Perfluorooctanoic acid	72%	78%	75%	8%
Perfluorononanoic acid	77%	78%	78%	1.3%
Perfluorodecanoic acid	15%	10%	12%	37%
Perfluorooctanesulfonic acid	73%	68%	70%	6.4%
Average Surrogate Recovery	62%	56%	59%	16%

NA - Not Applicable

Table S23 : Final Target Per- and polyfluoroalkyl Substances (PFAS) - Polyester Deployed for 3 Months - Channel Bottom

Matrix: Polyester (PET)

Date Extracted: 3/20/2019 & 5/6/2019 Sample Prep: Solid-Liquid Extraction

Data Analyzed: 07/01/2019 & 08/12/19 Instrument: Waters Quattro Micro LC-Tandem Mass Spectrometer

Identification	PET - Channel Bottom #1	PET - Channel Bottom #2	Average	%RPD
Units	ng/g	ng/g	ng/g	
**Native PFAS**				
Perfluorobutanesulfonic acid	0.078	< 0.05	0.078	NA
Perfluorohexanoic acid	< 0.05	< 0.05	< 0.05	NA
Perfluoroheptanoic acid	0.18	0.16	0.18	12%
Perfluorooctanoic acid	0.059	0.072	0.065	20%
Perfluorononanoic acid	0.14	0.25	0.20	57%
Perfluorodecanoic acid	< 0.05	<0.05	< 0.05	NA
Perfluorooctanesulfonic acid	< 0.05	<0.05	< 0.05	NA
Sum Target PFAS	0.38	0.48	0.43	24%
**Surrogate PFAS**	**%Recovery**	**%Recovery**	**Average**	**%RPD**
Perfluorobutanesulfonic acid	96%	92%	94%	3.7%
Perfluorohexanoic acid	119%	81%	100%	39%
Perfluoroheptanoic acid	99%	76%	87%	26%
Perfluorooctanoic acid	88%	70%	79%	23%
Perfluorononanoic acid	63%	56%	59%	13%
Perfluorodecanoic acid	29%	36%	32%	22%
Perfluorooctanesulfonic acid	60%	47%	53%	25%
Average Surrogate Recovery	79%	65%	72%	22%

NA - Not Applicable

Table S24 : Final Target Per- and polyfluoroalkyl Substances (PFAS) - Polyethylene Deployed for 3 Months - Lake Bottom

Matrix: Polyethylene (PE)
Date Extracted: 3/20/2019 & 5/6/2019
Sample Prep: Solid-Liquid Extraction
Data Analyzed: 07/01/2019 & 08/12/19
Instrument: Waters Quattro Micro LC-Tandem Mass Spectrometer

Identification	PE - Lake Bottom #1	PE - Lake Bottom #2	Average	%RPD
Units	ng/g	ng/g	ng/g	
**Native PFAS**				
Perfluorobutanesulfonic acid	< 0.05	< 0.05	< 0.05	NA
Perfluorohexanoic acid	0.076	< 0.05	0.076	NA
Perfluoroheptanoic acid	0.069	< 0.05	0.069	NA
Perfluorooctanoic acid	0.11	0.10	0.11	4.9%
Perfluorononanoic acid	< 0.05	< 0.05	< 0.05	NA
Perfluorodecanoic acid	< 0.05	<0.05	< 0.05	NA
Perfluorooctanesulfonic acid	< 0.05	0.069	0.069	NA
Sum Target PFAS	0.26	0.17	0.21	38%
**Surrogate PFAS**	**%Recovery**	**%Recovery**	**Average**	**%RPD**
Perfluorobutanesulfonic acid	97%	70%	84%	32%
Perfluorohexanoic acid	79%	71%	75%	11%
Perfluoroheptanoic acid	72%	72%	72%	0.0%
Perfluorooctanoic acid	91%	86%	89%	5.6%
Perfluorononanoic acid	120%	96%	108%	22%
Perfluorodecanoic acid	65%	52%	59%	22%
Perfluorooctanesulfonic acid	79%	72%	76%	9.3%
Average Surrogate Recovery	86%	74%	80%	15%

NA - Not Applicable

Table S25 : Final Target Per- and polyfluoroalkyl Substances (PFAS) - Polypropylene Deployed for 3 Months - Lake Bottom

Matrix: Polypropylene (PP)

Date Extracted: 3/20/2019 & 5/6/2019 — **Sample Prep: Solid-Liquid Extraction**

Data Analyzed: 9/23/219 — **Instrument: Waters Quattro Micro LC-Tandem Mass Spectrometer**

Identification	PP - Lake Bottom #1	PP - Lake Bottom #2	Average	%RPD
Units	ng/g	ng/g	ng/g	
**Native PFAS**				
Perfluorobutanesulfonic acid	< 0.05	< 0.05	< 0.05	NA
Perfluorohexanoic acid	< 0.05	< 0.05	< 0.05	NA
Perfluoroheptanoic acid	< 0.05	< 0.05	< 0.05	NA
Perfluorooctanoic acid	0.12	0.072	0.10	49%
Perfluorononanoic acid	< 0.05	< 0.05	< 0.05	NA
Perfluorodecanoic acid	< 0.05	< 0.05	< 0.05	NA
Perfluorooctanesulfonic acid	< 0.05	< 0.05	< 0.05	NA
Sum Target PFAS	0.12	0.072	0.10	49%
**Surrogate PFAS**	**%Recovery**	**%Recovery**	**Average**	**%RPD**
Perfluorobutanesulfonic acid	66%	77%	71%	15%
Perfluorohexanoic acid	64%	65%	64%	2.3%
Perfluoroheptanoic acid	60%	68%	64%	13%
Perfluorooctanoic acid	51%	70%	60%	32%
Perfluorononanoic acid	26%	32%	29%	21%
Perfluorodecanoic acid	16%	17%	16%	9.2%
Perfluorooctanesulfonic acid	41%	43%	42%	4.8%
Average Surrogate Recovery	46%	53%	50%	14%

NA - Not Applicable

Table S26 : Final Target Per- and polyfluoroalkyl Substances (PFAS) - Polyester Deployed for 3 Months - Lake Bottom

Matrix: Polyester (PET)

Date Extracted: 3/20/2019 & 5/6/2019 — Sample Prep: Solid-Liquid Extraction

Data Analyzed: 07/01/2019 & 08/12/19 — Instrument: Waters Quattro Micro LC-Tandem Mass Spectrometer

Identification	PET - Lake Bottom #1	PET - Lake Bottom #2	Average	%RPD
Units	ng/g	ng/g	ng/g	
**Native PFAS**				
Perfluorobutanesulfonic acid	< 0.05	< 0.05	< 0.05	NA
Perfluorohexanoic acid	0.062	0.059	0.060	5.4%
Perfluoroheptanoic acid	< 0.05	< 0.05	< 0.05	NA
Perfluorooctanoic acid	0.14	0.11	0.13	25%
Perfluorononanoic acid	< 0.05	< 0.05	< 0.05	NA
Perfluorodecanoic acid	< 0.05	< 0.05	< 0.05	NA
Perfluorooctanesulfonic acid	< 0.05	< 0.05	< 0.05	NA
Sum Target PFAS	0.20	0.17	0.19	19%
**Surrogate PFAS**	**%Recovery**	**%Recovery**	**Average**	**%RPD**
Perfluorobutanesulfonic acid	113%	136%	124%	19%
Perfluorohexanoic acid	126%	119%	122%	5.3%
Perfluoroheptanoic acid	110%	98%	104%	12%
Perfluorooctanoic acid	81%	81%	81%	0.6%
Perfluorononanoic acid	41%	50%	46%	20%
Perfluorodecanoic acid	13%	15%	14%	18%
Perfluorooctanesulfonic acid	30%	45%	37%	41%
Average Surrogate Recovery	73%	78%	75%	16%

NA - Not Applicable

Table S27: Per- and polyfluoroalkyl Substances (PFAS) Quality Control Summary - 3-Month Deployed Plastics

Identification		-	3-Month PP Lake Bottom	3-Month PE Channel Bottom	3-Month PET Lake Bottom
	ng/g	%Recovery	%Recovery	%RPD	%Recovery
	Reagent Blank	Reagent Blank Spike	Analytical Spike	Analytical Sample Duplicate	Sample Matrix Spike
Perfluorobutanesulfonic acid	< 0.05	108%	129%	NC1	121%
Perfluorohexanoic acid	< 0.05	101%	92%	18%	109%
Perfluoroheptanoic acid	< 0.05	103%	85%	0.0%	110%
Perfluorooctanoic acid	< 0.05	110%	77%	30%	121%
Perfluorononanoic acid	< 0.05	141%	71%	NC1	134%
Perfluorodecanoic acid	<0.05	106%	78%	NC1	81%
Perfluorooctanesulfonic acid	<0.05	105%	93%	NC1	85%
**Average Recovery & RPD**	NA	111%	89%	16%	109%
**Standard Deviation**	NA	14%	19%	15%	20%

NA - Not Applicable

NC1 - Not calculated, less than detection limit.
